# Supplementary material for: Unraveling the dynamics of conductive filaments in MoS2-based memristors by operando transmission electron microscopy
Source: Nat Commun. 2025 Aug 12;16:7433. doi: 10.1038/s41467-025-62592-2 (PMC12343884; doi:10.1038/s41467-025-62592-2)
Supplement: Supplementary file 1 — Supplementary Information [file 41467_2025_62592_MOESM1_ESM.pdf]

Supplementary Information for

Unraveling the dynamics of conductive filaments in MoS_2 -based memristors by *operando* transmission electron microscopy

Ke Ran[1,2,3*], Janghyun Jo[3], Sofía Cruces[4], Zhenxing Wang[1], Rafal E. Dunin-Borkowski[3], Joachim Mayer[2,3] and Max C. Lemme[1,4*]

[1] Advanced Microelectronic Center Aachen, AMO GmbH, Aachen, Germany

[2] Central Facility for Electron Microscopy GFE, RWTH Aachen University, Aachen, Germany

[3] Ernst Ruska-Centre for Microscopy and Spectroscopy with Electrons ER-C, Forschungszentrum Jülich GmbH, Jülich, Germany

[4] Chair of Electronic Devices ELD, RWTH Aachen University, Aachen, Germany

* Correspondence: ran@amo.de, lemme@amo.de

Supplementary Note 1: The active Ag electrode

Supplementary Fig. 1 compares the same lamella before and after exposure to air and light for 5 days (additional FIB milling was applied in between). Here, instead of Al (as shown in Fig. 1a), a Pd layer of ~50 nm is deposited on top of the Ag electrode for protection. Supplementary Fig. 1a-c show the TEM images and EDXS elemental mapping results from the freshly prepared lamella. For lamellas, the Pd will not be able to provide the same protection for Ag as in the bulky device, since a large surface of Ag is unavoidably exposed after FIB preparation. After the lamella was stored in air without any protection for 5 days, TEM investigations were taken and are shown in Supplementary Fig. 1d-f. Significant changes can be observed for the Ag layer, which seems to have expanded. According to the EDXS elemental mapping in Supplementary Fig. 1f, strong S signal coincides with Ag, suggesting a possible reaction between Ag and S from MoS_2 by exposing the lamella to air and light. Thus, to avoid such structural degradations, all the lamellas in our work were prepared shortly before the *operando* TEM experiments, always kept in a desiccator, and protected from light.

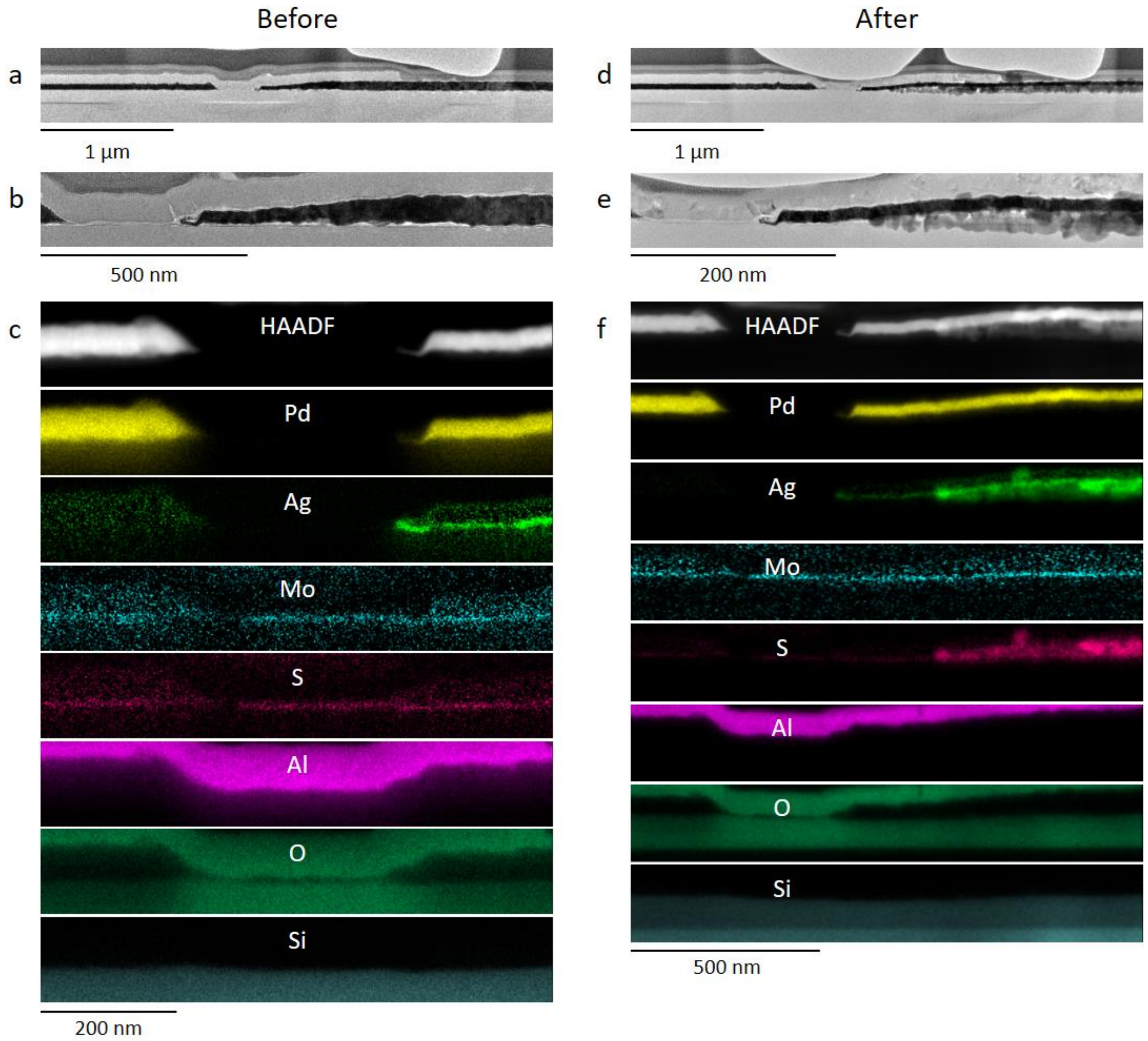

Supplementary Figure 1: TEM images (a-b) and EDXS elemental mapping results (c) of a freshly prepared lamella. (d-f) The corresponding results from the same lamella after 5 days in air and exposure to light.

Supplementary Note 2: Detailed structure of the lamella in Fig. 1e-g (L1)

Supplementary Fig. 2a-c show bright field scanning transmission electron microscopy (BF STEM) images of the lamella, as shown in Fig. 1e-g (L1). The bright layer above the electrodes is the extra deposited Al_2O_3, which is ~6.6 µm wide and ~80 nm thick in the z direction, fully covering the channel (~0.8 µm wide) between the two electrodes. The dark layer above the Al_2O_3 is Pt from the FIB preparation. For later *operando* measurements, a Pt-free region and an Al_2O_3-free region were achieved via careful FIB milling, as shown in Supplementary Fig. 2b. This allowed a direct contact between the Au tip and the Pd electrode, as shown in Supplementary Fig. 2c. Supplementary Fig. 2d-e show the TEM image and thickness map recorded from L1. The thickness map shows the t/λ ratio (t: sample thickness in the y direction and λ: electron inelastic mean free path) of the imaged region. From the SiO_2 region next to the MoS_2 bundles, a ratio of ~0.6 was estimated. Thus, the thickness of the channel in the y direction can be roughly estimated to be less than 100 nm[1].

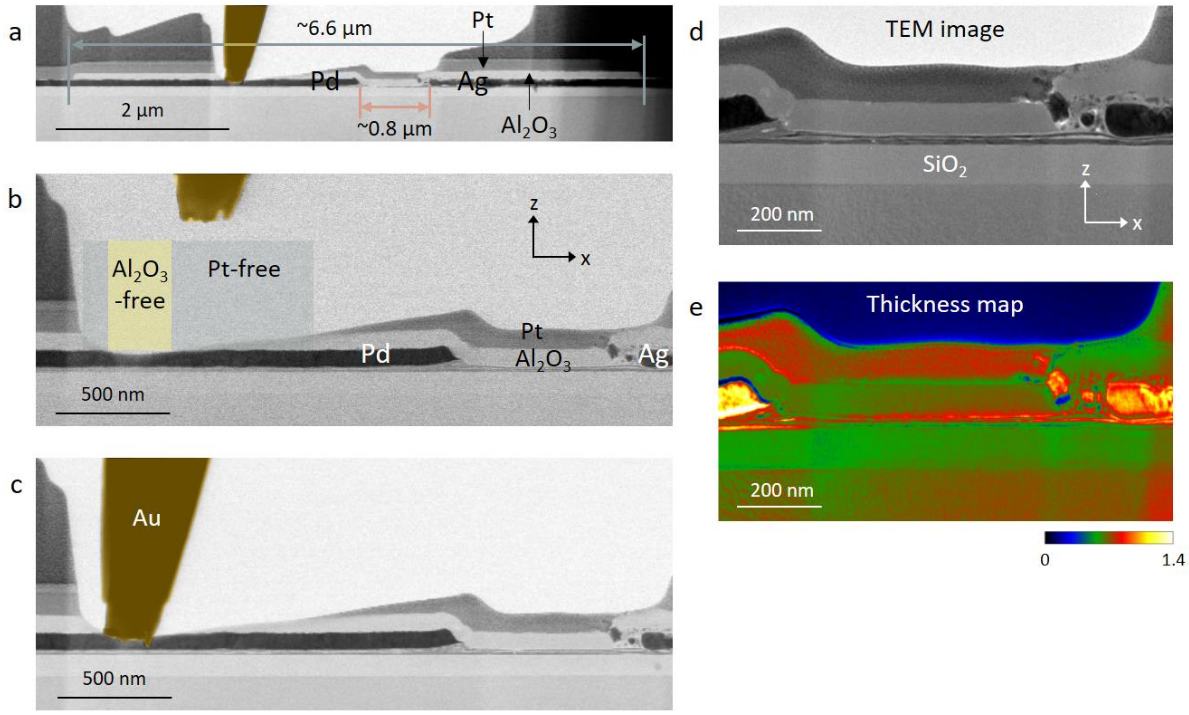

Supplementary Figure 2: (a-c) BF STEM images of L1, showing the additionally deposited Al_2O_3 layer (a), the FIB fabrication of Pt-free and Al_2O_3-free region (b), and the direct contact between Au tip and the Pd electrode. (d-e) TEM image and a thickness map recorded around the channel to estimate the sample thickness in the y direction.

Supplementary Note 3: EDXS elemental mapping results in Fig. 1g and 2d

Supplementary Figs. 3a-b show the EDXS elemental mapping results for all the elements, corresponding to Fig. 1g and Fig. 2d. Some Pt from the FIB preparation is observed between Pd and MoS_2, as outlined by the solid rectangles. However, these penetrated Pt molecules were rather stable during the *operando* experiment and thus should have negligible effects on resistive switching. Between the two EDXS elemental mappings in Supplementary Fig. 3a and 3b, a bias voltage sweep of 0 V → -5 V → 0 V was applied, as shown in Supplementary Fig. 3d, inducing nonvolatile switching and leaving the device at LRS. Supplementary Fig. 3c compares the two composite maps from Supplementary Fig. 3a-b. As outlined by the circles, the small tail of the Pd electrode disappears after biasing, as does the Ag particle close to the Ag electrode on the right. Thus, the tail of Pd could be active for resistive switching, due to its small size and large surface. However, such Pd switching was only observed once at the beginning, and the Pd electrode was rather stable during all the following biasing sweeps (the constant ΔA1 in Fig. 3b). The marked Ag particle on the right might just loosely lie on the surface of the lamella (in the y direction) and thus can be easily removed during biasing (by the generated heat and/or mechanical instability).

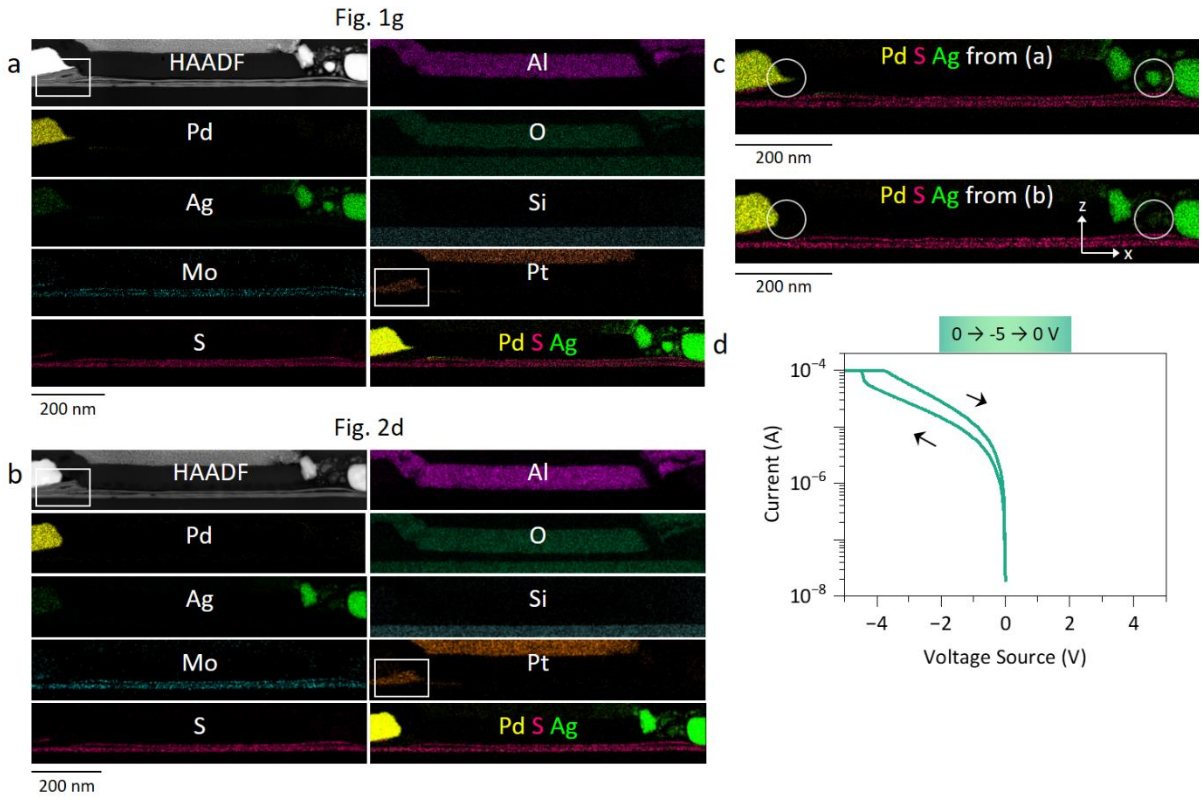

Supplementary Figure 3: (a-b) EDXS elemental mapping results for all the elements, corresponding to Fig. 1g and Fig. 2d. (c) Comparison of the composite maps from (a-b). (d) *I-V* curve from a biasing sweep 0 V → -5 V → 0 V applied between (a) and (b).

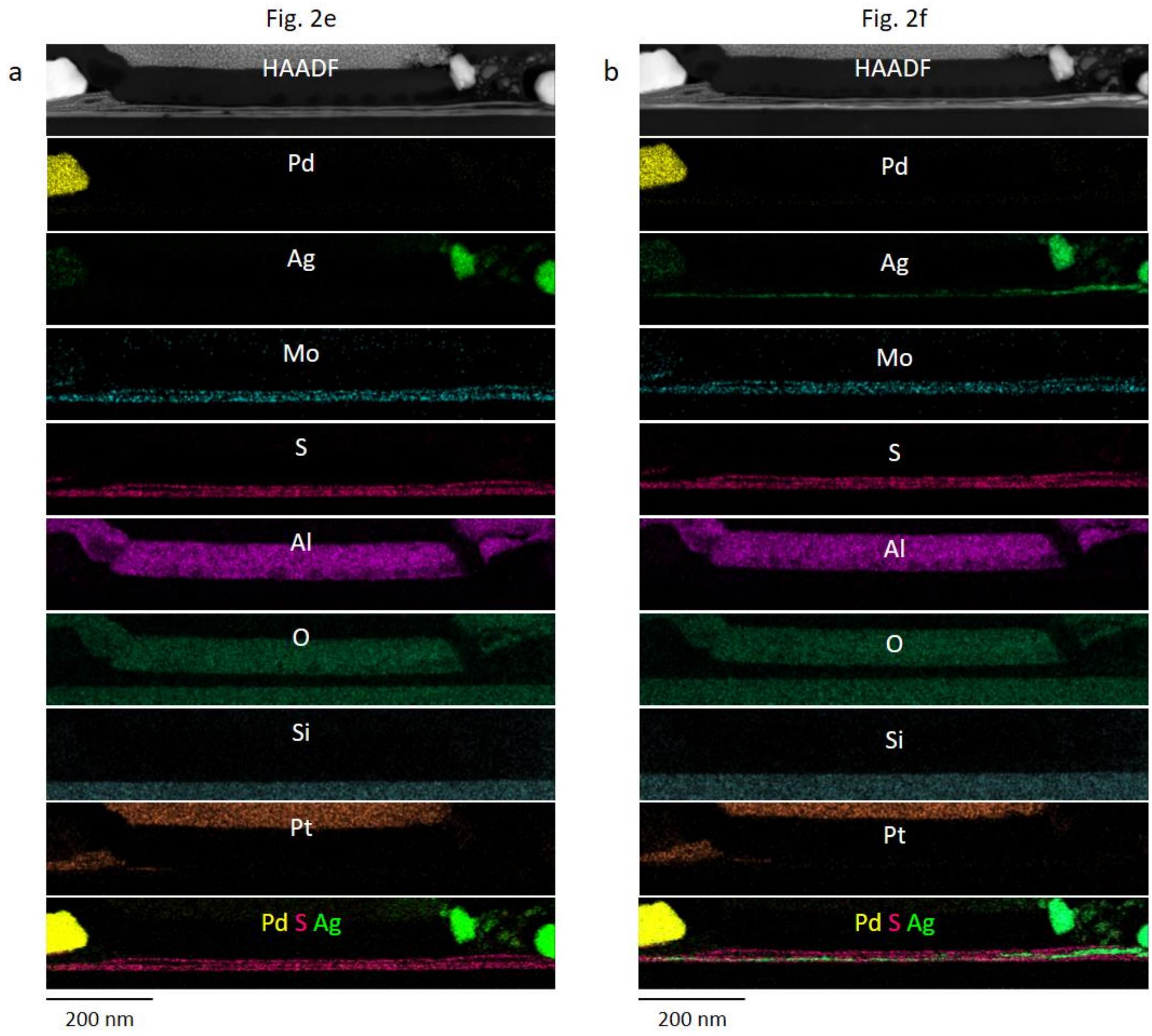

Supplementary Figure 4: (a-b) EDXS elemental mapping results for all the elements, corresponding to Fig. 2e-f.

Supplementary Note 4: Composition of the filaments

To confirm the composition of the filaments, EDX spectrum from local region is extracted and plotted in Supplementary Fig. 5 (the top row). Supplementary Fig. 5a is reproduced from Fig. 2f, and the dashed rectangles in Supplementary Fig. 5c defined the region for the spectrum in Supplementary Fig. 5e. In addition to the Cu K peak (from the TEM grid), a strong Ag L peak together with a small S K peak show up (see the inset for details), suggesting that the filaments are mainly composed of Ag. To understand the origin of the small S K peak, EDX spectrum from the same region before the RS is also plotted in Supplementary Fig. 5 (the bottom row). Supplementary Fig. 5b is reproduced from Fig. 2d, and the dashed rectangles in Supplementary Fig. 5d defined the region for the spectrum in Supplementary Fig. 5f. There, without any detectable Ag signal, a small S K peak shows up again. Moreover, the heights of both S K peaks in Supplementary Fig. 5e and 5f are comparable. Thus, the small S peak in Supplementary Fig. 5e can be safely attributed to the surrounding MoS_2 (most likely due to delocalization of electron beam during EDXS elemental mapping), and the filaments detected in our case are pure Ag.

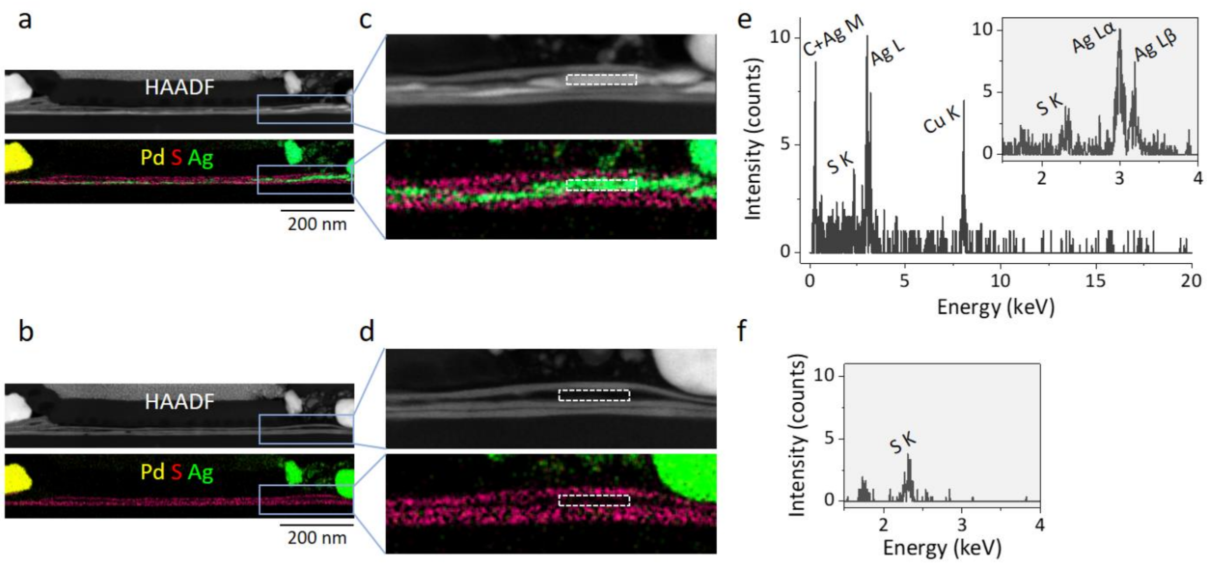

Supplementary Figure 5: (a-b) Reproductions from Fig. 2f and 2d. (c-d) Enlarged HAADF image and composite map from the regions outlined by the rectangles in (a-b). (e) EDX spectrum extracted from the region outlined by the dashed rectangle in (c). Inset shows details around the Ag peak. (f) EDX spectrum extracted from the region outlined by the dashed rectangle in (d).

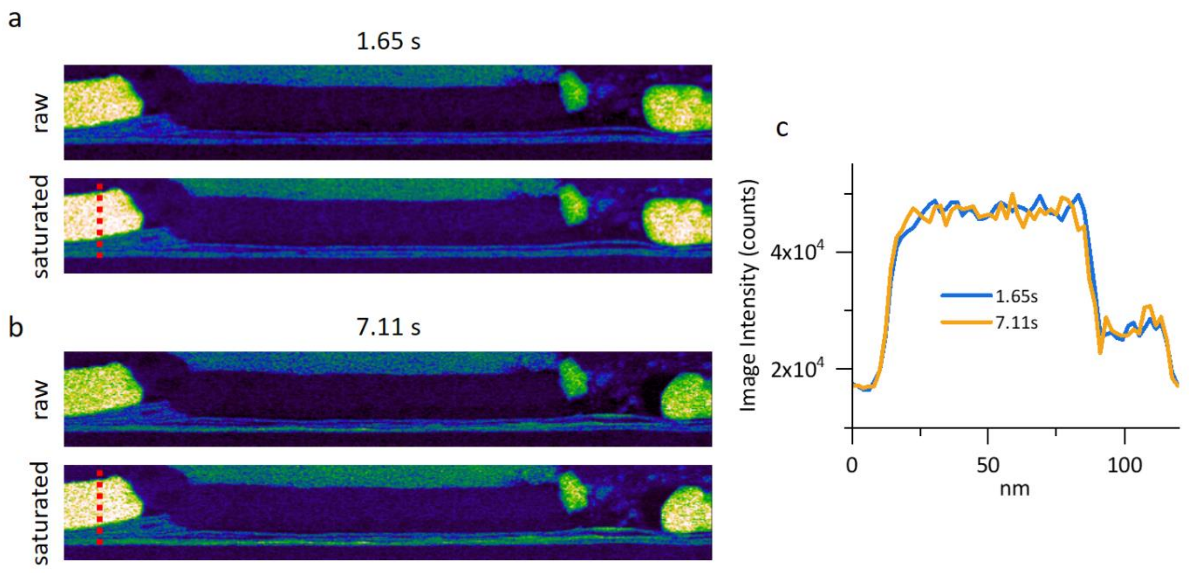

Supplementary Figure 6: (a-b) Comparison of the raw images with the saturated images in Figure 3a at 1.65 s and 7.11 s, respectively. While the raw images were recorded within a sufficient dynamic range to monitor the intensity variation during resistive switching, the images shown in Fig. 3a were saturated to emphasize the Ag within the channel (the same applies to Fig. 4h, Fig. 5m-p). (c) Intensity line profiles along the dotted lines in (a-b).

Supplementary Note 5: Lamella in Fig. 4a-d and Fig. 4h (L2)

The lamella used for Fig. 4a-d and Fig. 4h (L2) is shown in Supplementary Fig. 7a. The channel between the two electrodes is ~0.8 µm wide. It is fully covered by an Al_2O_3 layer, which is ~5.4 µm wide. On the side of the Pd electrode, the Pt layer from the FIB preparation and the Al_2O_3 layer were partially removed to enable a direct contact between the Au tip and the Pd. Obvious defects were observed in the MoS_2 channel, as indicated by the arrows in Supplementary Fig. 7b. The MoS_2 layers are locally broken, and the number of MoS_2 layers varies between 5 and 10, as shown in Supplementary Fig. 7c. Based on the thickness map in Supplementary Fig. 7e, a t/λ ratio of ~0.6 was estimated from the SiO_2 region next to the MoS_2, suggesting that the thickness of the channel region is comparable to that of L1 (<100 nm).

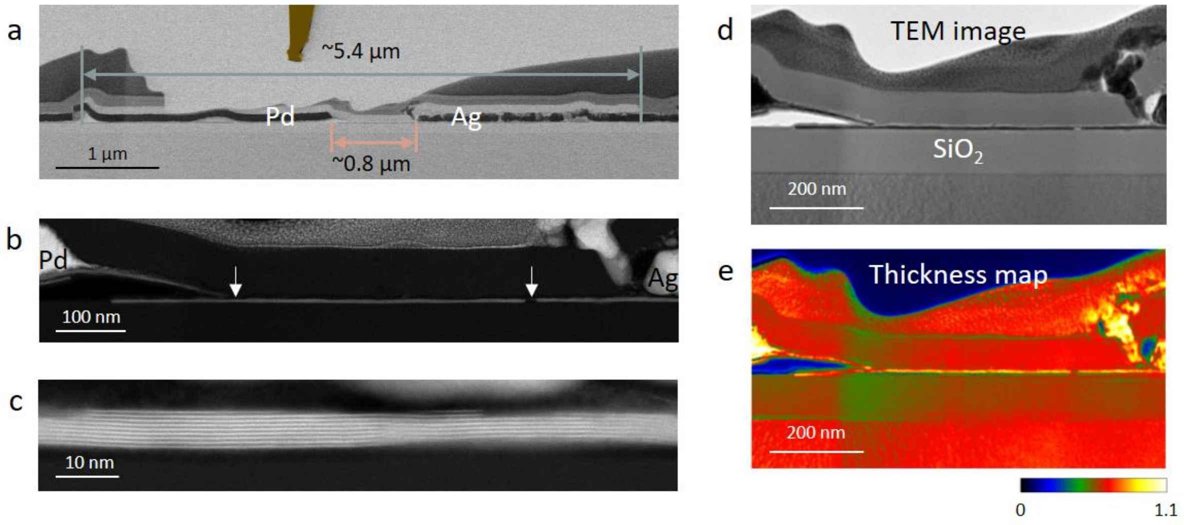

Supplementary Figure 7: (a) BF STEM image of L2. (b-c) HAADF images of L2, showing an overview of the channel and the local structure of the single MoS_2 bundle. (d-e) TEM image and a thickness map recorded around the channel.

Supplementary Note 6: Bias voltage sweep applied to L2 for Fig. 4a-d

Supplementary Fig. 8b shows the *I–V* response of L2 under a 0 V → 10 V → 0 V → -10 V → 0 V sweep, corresponding to Fig. 4a-d, where nonvolatile switching was induced. Several images from the image series are shown in Supplementary Fig. 8a.

During RS, the formed Ag filaments will intercalate into the vdW gap of MoS_2, and cause expanded D. By analyzing the FFT from the section of multilayer MoS_2, in Supplementary Fig. 8a, the evolution of D_m during a successful RS was monitored, and an expansion of D_m up to 10% was determined. Moreover, we integrated the intensities of the whole field of view and plotted the intensity variation as a function of time in Supplementary Fig. 8c. Although the changes are much lower than ΔA2 in Fig. 3b, an intensity increase of 5% is still noticeable in the LRS. Compared with Supplementary Fig. 8d, which is reproduced from Fig. 4d, the intensity variation and ΔD_m vary coincidently. Thus, based on the successful RS, and the coincident evolutions of image intensity (mostly likely caused by the emerging Ag CFs) and D, the conclusion that vdW gaps host the Ag migration and contribute to the RS can be made.

It should be noted, that the averaged ΔD_m in Fig. 4d may well below its local maxima. To estimate ΔD_m, all the local expansions of D were averaged within the field of view in Fig. 4a, and also along the direction of electron beam for ~100 nm (thickness of the lamella). For example, as illustrated in Supplementary Fig. 8e, during a successful RS, Ag chains may form only in one of the 6 vdW gaps in Supplementary Fig. 8a. Thus, the local ΔD will be divided by 6 to estimate the averaged ΔD_m. Besides, as suggested in Supplementary Fig. 8f, within the xy plane, the formed Ag chains may be randomly distributed. While the electrons are travelling along the y direction, the imaged ΔD is also an averaged value over the ~100 nm along y direction. Thus, in order to quantitatively evaluate the intercalation process, numerical calculations are necessary. Details, such as the activation energy for the Ag intercalation, the maximum capacity of each vdW gap for Ag ions, the caused reversible/irreversible structural deformation of MoS_2, and possible bonding between Ag and S ions are all critical information to advance our understanding of the switching mechanism.

Supplementary Fig. 8g illustrates the proposed ECM mechanism[2]: (A) oxidation and dissolution of the active electrode (Ag/Cu) under positive bias; (B) migration of Ag/Cu ions; (C) reduction at the inert electrode; (D-E) nucleation and filamentary growth; and (F) the final transition from HRS to LRS.

It should be also noted, although individual MoS_2 layers can be resolved in Supplementary Fig. 8a, no significant intensity from Ag within the vdW gaps can be detected. Along the e-beam direction (y direction as defined in Fig. 1e), the MoS_2 is ~100 nm thick, while the formed Ag filaments can be as thin as 2.52 Å. Such significant difference in the amounts of Mo and Ag could primarily account for the unrecognizable Ag contrast in Supplementary Fig. 8a, together with the noisy HAADF images due to short frame time and the possibly insufficient temporal resolution.

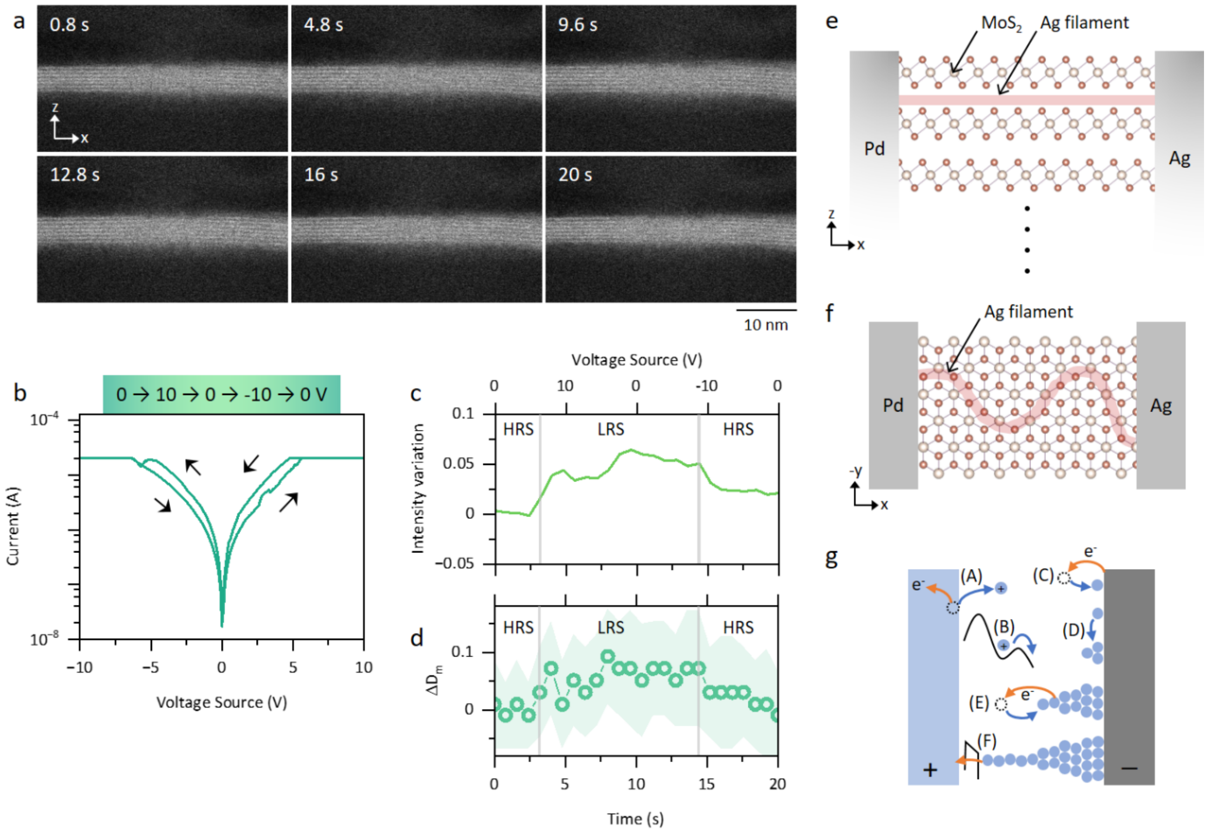

Supplementary Figure 8: (a) HAADF images recorded at different times during the sweep in (b), corresponding to Fig. 4a-d. (c) Intensity variation of the images as a function of time. (d) A reproduction from Fig. 4d. The corresponding bias voltage is indicated on the top axis. (e) Illustration of Ag chain only formed within one vdW gap of the multilayer MoS_2. (f) Possible distribution of the Ag chain within the xy plane. Electron beam/viewing direction is along the y direction. (g) The proposed ECM mechanism[2].

Supplementary Note 7: The Ag intercalation into bundle #3 in Fig. 4f

To confirm the Ag intercalation into bundle #3, the same region of MoS_2 before and after the RS was compared. Supplementary Fig. 9a is reproduced from Fig. 1f, where L1 was at HRS before applying any bias voltage. The middle region, as outlined by the rectangle in blue is further enlarged in Supplementary Fig. 9b, and the same region was imaged again after RS at LRS in Supplementary Fig. 9c (a reproduction of Fig. 4f). In Supplementary Fig. 9b, three bundles can be clearly distinguished. The contrast change along each bundle (the parallel direction) is most likely due to the channeling effect caused by the different orientations of each MoS_2 layers within the bundle. As denoted by the red arrows, around the bright peaks in Supplementary Fig. 9c, there was no gap with ~3 nm width detected in Supplementary Fig. 9b before the RS. As also demonstrated in Fig. 2g, the emerging strong image contrast after RS can be directly associated with the formation of Ag CFs. Thus, the ~3 nm-wide bright peaks can be attributed to the Ag intercalation into the vdW gaps of MoS_2, instead of into the gaps between MoS_2 bundles (the case outlined by the rectangles in red).

It should also be noted, that Supplementary Fig. 9a was recorded with a much lower spatial resolution compared with Supplementary Fig. 9c, to include the whole channel of L1 into the field of view. During the experiment, unnecessary high-resolution imaging was always avoided, as MoS_2 is rather sensitive to the electron beam, and structural damages can be easily induced. Nevertheless, the comparisons between Supplementary Fig. 9b and 9c confirm that the Ag intercalated into bundle #3, resulting in the strong and broad peaks in Fig. 4g, as well as a ~11% expansion of the vdW gap.

It is also noticed, that the contrast from Ag in Fig. 4g is much more significant than that in Fig. 4a, although comparable expansions of D of ~10% were estimated for both cases. As illustrated in Supplementary Fig. 9d-e, this can be explained by the excess Ag going out of the MoS_2 through its open edges, and landing on the surface of the FIB lamella. During RS, the formed Ag filaments can travel freely within the xy plane. When excess Ag filaments are formed, i.e. more than what can be accommodated by the vdW gaps of the MoS_2, one option

for them is to go outside of the MoS_2 through its open edges, and accumulate on the surface, see Supplementary Fig. 9e. For HAADF imaging, the electron beam is coming from the y direction, and the resulted image contrast is proportional to the amount of materials along the y direction. Therefore, both the Ag filaments within the vdW gap and the excess Ag landing on the surface are contributing to the ~3 nm wide strong peaks in Fig. 4g. For the case of Fig. 4a, we would expect much less Ag CFs formed during RS (the *I-V* curves in Supplementary Fig. 8b and in Fig. 2c), and thus much less excess Ag landing on the surface.

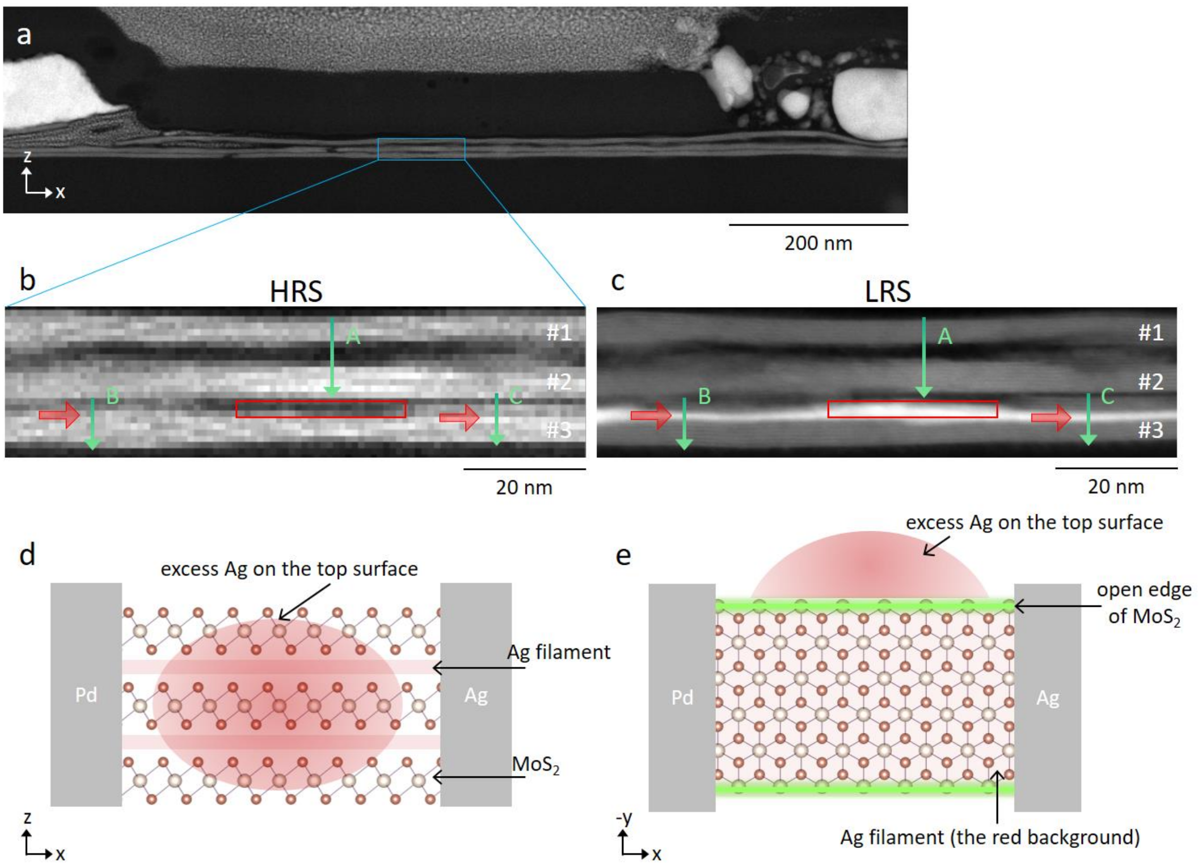

Supplementary Figure 9: (a-b) The HAADF image reproduced from Fig. 1f, and an enlarged image from the middle region outlined by the rectangle. (c) A reproduction from Fig. 4f. The arrows and rectangles mark the same locations in (b-c). (d) Cross-sectional view of the device at LRS, with Ag filaments within the vdW gaps of MoS_2, as well as excess Ag on the top surface. (e) Plan-view of the device illustrated in (d). Here, the Ag filaments are filling the vdW gaps uniformly for simplicity, and the electron beam is coming along y direction.

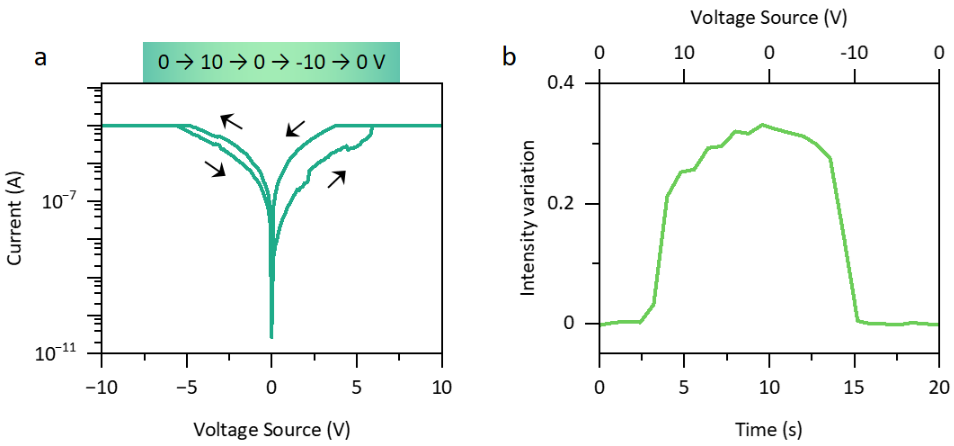

Supplementary Figure 10: (a) *I*-*V* curve corresponding to Figure 4h. (b) Intensity variation of the image as a function of time. The corresponding bias voltage is indicated on the top axis.

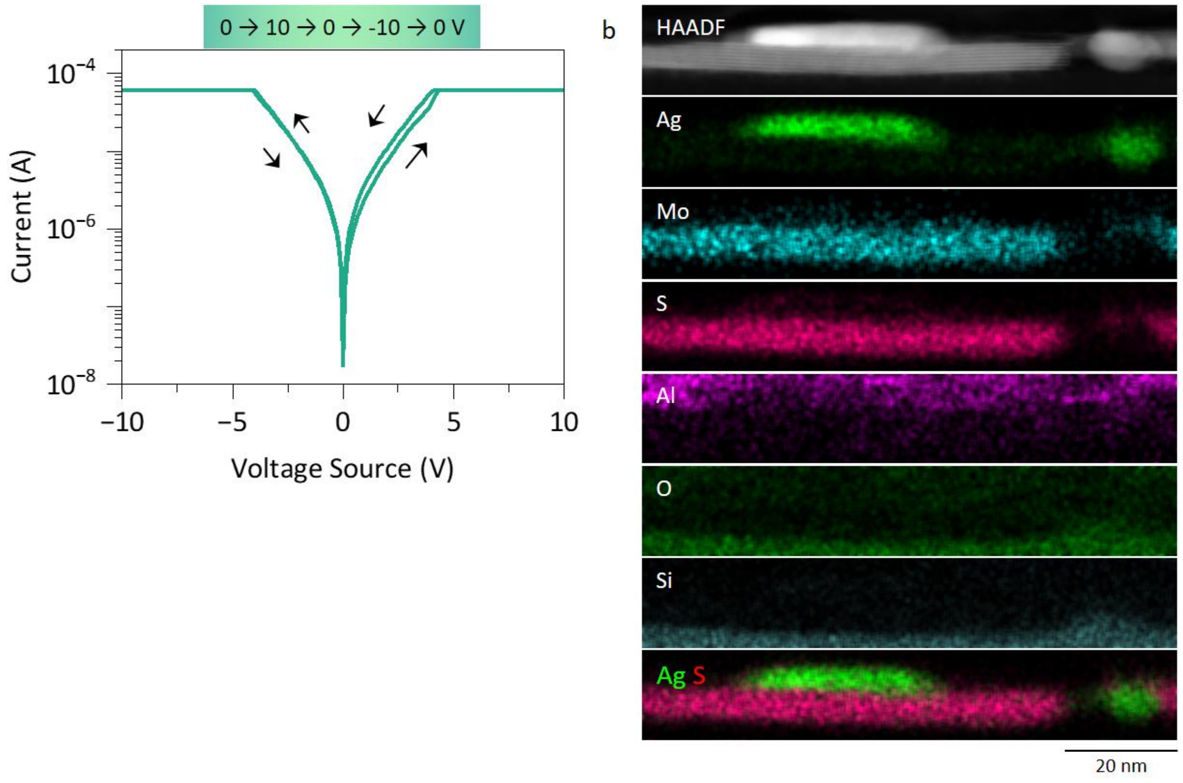

Supplementary Figure 11: (a) *I*–*V* curve from a biasing sweep of 0 V → 10 V → 0 V → -10 V → 0 V applied to L2, where the RESET failed. (b) EDX elemental mapping results from a local region within the channel after the sweep in (a). Significant Ag residuals are observed at the top surface of the MoS_2 and at the broken part of the MoS_2.

Supplementary Note 8: Removing the surface contamination

The negative ΔA2 noticed in Fig. 5e, 5h, and 5k can be attributed to the removal of contaminations at the lamella surface during the initial bias sweeps. As shown in Supplementary Fig. 12a, one major type of these contaminations is the redeposited Ag from the FIB preparation (the sparse Ag signals as denoted by the arrow), which loosely lies on the surface of the lamella before electrical measurement. After one bias voltage sweep (Supplementary Fig. 3d), these Ag signals together with the large Ag particle outlined by the circle all disappeared in Supplementary Fig. 12b, leading to a reduced image contrast thus a negative ΔA2. Besides, hydrocarbon contaminations were also expected in our lamellas, since no plasma clean has been applied (oxygen is involved during the cleaning which could potentially react with the Ag electrode). During STEM imaging, the residual hydrocarbon contaminations will accumulate to the area irradiated by the electron beam and cause extra bright contrast. However, we didn't observe such feature even during imaging with high resolution. Thus, most of the hydrocarbon contaminations should be already removed during the initial bias voltage sweeps, contributing to the reduced image intensity as well.

The removal of these contaminations could be caused by the heat generated during the RS. Using a very approximate physical model, the temperature of the device during operation can be estimated. Taking L1 for example, the length and width of the MoS_2 conductive channel are $L \approx 800$ nm and $W \approx 100$ nm, and the thickness of MoS_2 is $t \approx 20$ nm (Supplementary Note 2 and Supplementary Fig. 2). The mass density of multi-layer MoS_2 is about $\rho = 5.06\times10^6$ g/m^3.[3] Therefore, a total mass of MoS_2 of $m = \rho\cdot L\cdot W\cdot t = 8.1\times10^{-15}$ g is obtained. The specific heat capacity (per unit cell) at room temperature (300 K) of MoS_2 is $C_m = 0.3$ J·g^{-1}·K^{-1}, which can be expressed by

$$C_m = (Q_j - Q_c)/(m\cdot\Delta T), \qquad \text{Eq. 1}$$

where Q_j is the joule heat from the switch cycle (1.75×10^{-3} J based on the *I-V* curve $\int I\cdot V\cdot dt$, taking the curve in Fig. 2b for example), and Q_c is the heat conducted away through MoS_2. For simplicity, the highest temperature is assumed at the center of the MoS_2, and the generated

heat is dissipating from there to both electrodes which are at room temperature (300 K). ΔT is the temperature difference between the center of the MoS_2 channel and the electrodes.

The in-plane thermal conductivity of MoS_2 is $\kappa = 44\ W \cdot m^{-1} \cdot K^{-1}$.[4] Assuming the time span is roughly the same as the cycle time (~10 sec), κ can be expressed by

$$\kappa \cdot 10\ \text{sec} = Q_c \cdot (L/2)/(W \cdot t \cdot \Delta T) \qquad \text{Eq. 2}$$

Solving Eq.1 and Eq.2 together, $\Delta T = 795$ K is estimated, resulting in ~1065 K at the center of the MoS_2 channel, which is in line with the reports in literature[5,6].

However, it should be noted that this rough calculation is based on significant simplifications of the actual system. The parameters of MoS_2 (such as C_m and κ) are actually temperature-dependent, and the estimated ΔT is only an averaged value over one bias sweep. In order to accurately assess the evolution of device temperature during RS, dedicated numerical calculation using finite element method is necessary.

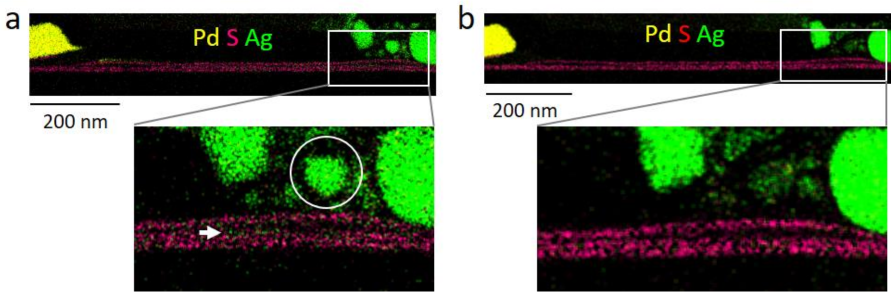

Supplementary Figure 12: (a-b) Reproductions from Fig. 1g and Fig. 2d, respectively. One bias voltage sweep was between the two maps.

Supplementary Note 9: Monitoring the biasing sweep applied to L2

A 0 V → 6 V → 0 V → -6 V → 0 V bias voltage sweep with 0.06 V/step, 0.05 s/step, and CC = 10^{-5} A was applied to L2, inducing nonvolatile switching. Supplementary Fig. 13a shows the corresponding *I-V* curves. Simultaneously, a series of HAADF images with a frame time of 0.22 s were recorded, and selected images are shown in Supplementary Fig. 13b. Despite successful switching, and unlike Fig. 3a, no significant changes can be directly observed in the images, suggesting limited formation of Ag filaments and a stable Ag electrode. Three regions are defined in the top image of Supplementary Fig. 13b, A2 (the channel as a whole), A2' (around a broken part of the MoS_2), and A4 (the Ag electrode). The intensity variations within the three regions are then plotted as a function of time in Supplementary Fig. 13c, with the bias voltage indicated at the top. The ΔA4 curve is rather noisy, close to zero and relatively stable without a clear pattern, indicating negligible Ag loss during resistive switching. In contrast, ΔA2 and ΔA2' behave quite similarly, except for the much larger values estimated from ΔA2'. Thus, the variations along A2 are caused mainly by A2' but are largely reduced after averaging within the whole channel. The right column of Supplementary Fig. 13b shows enlarged images of A2' at different times. Within this small region, certain intensity variations can be distinguished, corresponding to the peaks in Supplementary Fig. 13c, as marked by the asterisks. Overall, much less Ag is involved in the resistive switching of L2 than in the case with L1.

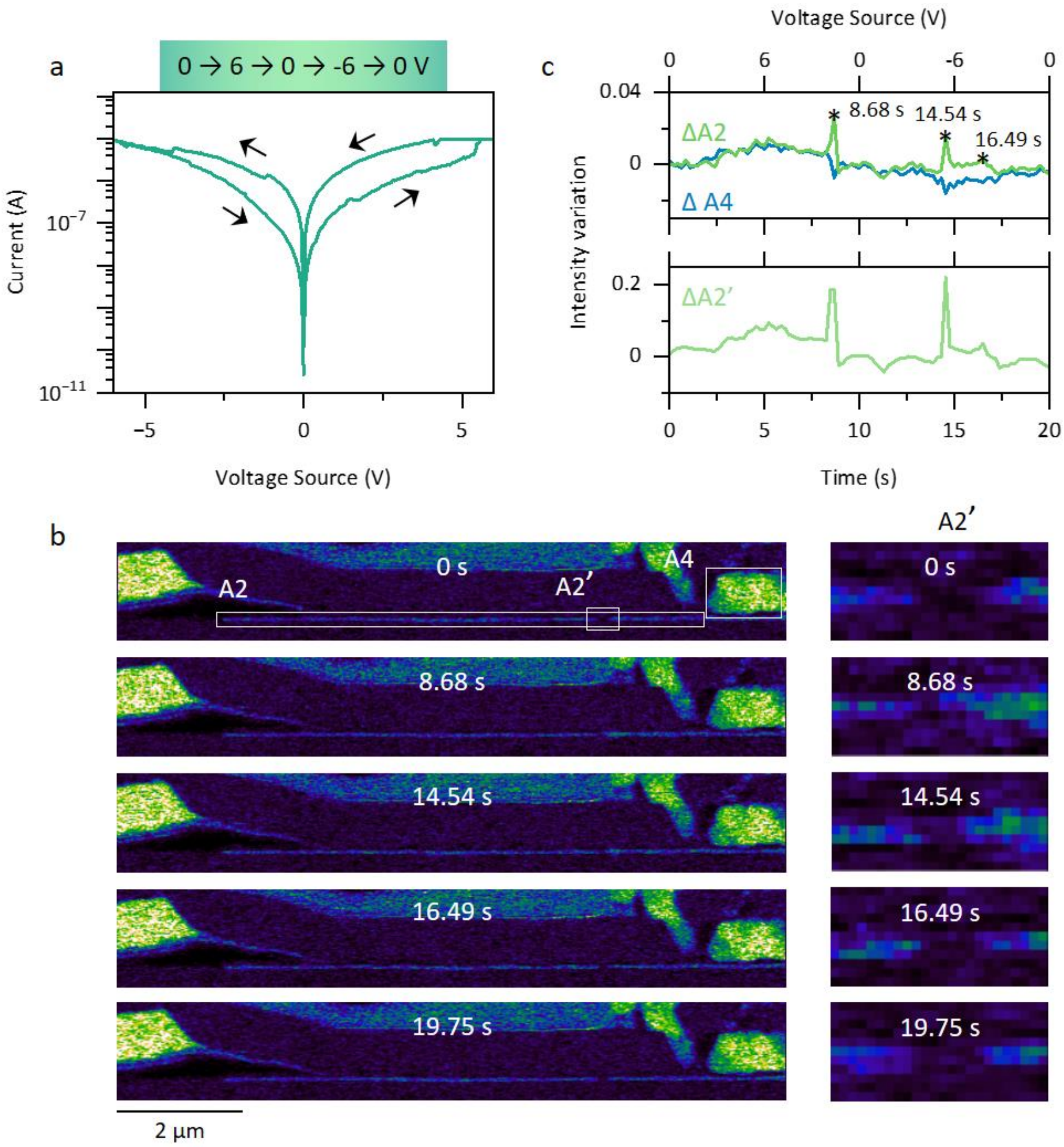

Supplementary Figure 13: (a) *I*–*V* response from L2 under a 0 → 6 → 0 → -6 → 0 V bias sweep. (b) Selected images from the simultaneously recorded HAADF image series at different times. Three regions are defined by the dotted rectangles: A2, A2', and A4. The enlarged image from A2' at different times is shown on the right. (c) The estimated intensity variations from A2, A2', and A4. The corresponding bias voltage is indicated on the top axis.

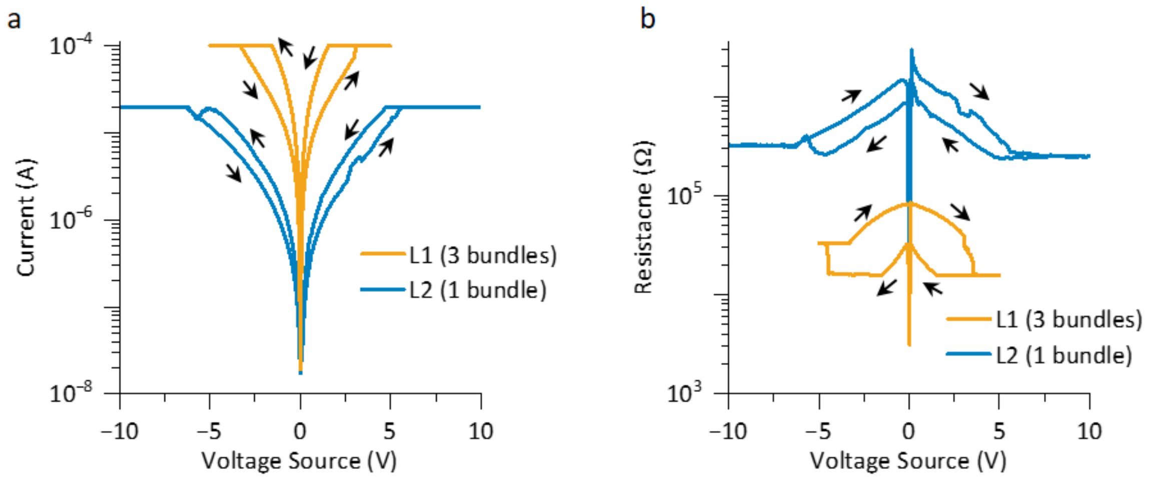

Supplementary Figure 14: Comparing the resistive switching between L1 and L2.

Supplementary References

1. Lee, C., Ikematsu, Y. & Shindo, D. Measurement of mean free paths for inelastic electron scattering of Si and SiO2. *J. Electron Microsc. (Tokyo)* **51**, 143–148 (2002).
2. Menzel, S., Tappertzhofen, S., Waser, R. & Valov, I. Switching kinetics of electrochemical metallization memory cells. *Phys. Chem. Chem. Phys.* **15**, 6945–6952 (2013).
3. Thomas, N. *et al.* 2D MoS2: structure, mechanisms, and photocatalytic applications. *Mater. Today Sustain.* **13**, 100073 (2021).
4. Bae, J. J. *et al.* Thickness-dependent in-plane thermal conductivity of suspended MoS2 grown by chemical vapor deposition. *Nanoscale* **9**, 2541–2547 (2017).
5. Teja Nibhanupudi, S. S. *et al.* Ultra-fast switching memristors based on two-dimensional materials. *Nat. Commun.* **15**, 2334 (2024).
6. Yang, S. J. *et al.* Volatile and Nonvolatile Resistive Switching Coexistence in Conductive Point Hexagonal Boron Nitride Monolayer. *ACS Nano* **18**, 3313–3322 (2024).
