# Supplementary material for: Unraveling the dynamics of conductive filaments in MoS2-based memristors by operando transmission electron microscopy
Source: Nat Commun. 2025 Aug 12;16:7433. doi: 10.1038/s41467-025-62592-2 (PMC12343884; doi:10.1038/s41467-025-62592-2)
Supplement: Supplementary file 2 — Description of Additional Supplementary Files [file 41467_2025_62592_MOESM2_ESM.pdf]

Description of Additional Supplementary Files

File name: Supplementary Video 1
Description: Image series corresponding to Fig. 3a. Frame time 0.127 s, and field of view 1052 nm by 155 nm.

File name: Supplementary Video 2
Description: Image series corresponding to Fig. 4a. Frame time 0.8 s, and field of view 42 nm by 42 nm.

File name: Supplementary Video 3
Description: Image series corresponding to Fig. 4h. Frame time 0.8 s, and field of view 23 nm by 10.8 nm.

File name: Supplementary Video 4
Description: Image series corresponding to Fig. 5. Frame time 0.127 s, and field of view 1052 nm by 155 nm.
